# Supplementary material for: Impact of Universal Test and Treat (UTT) on anticipated stigma among patients newly diagnosed with HIV in Johannesburg, South Africa: A cross-sectional study
Source: PLOS Glob Public Health. 2026 Jan 27;6(1):e0004779. doi: 10.1371/journal.pgph.0004779 (PMC12843591; doi:10.1371/journal.pgph.0004779)
Supplement: S1 Text — (DOCX) [file pgph.0004779.s001.docx]

*ART refusal cohort study*

Study ID __________________________________

**INTERVIEWER READS: NOW I WOULD LIKE TO ASK YOU SOME QUESTIONS ABOUT HOW YOU FEEL ABOUT TELLING OTHER PEOPLE ABOUT YOUR HIV STATUS.**

1. **PERCEPTIONS ABOUT DISCLOSURE OF HIV STATUS**

Strongly agree Agree Disagree Strongly disagree

- 1. I worry that people may judge me when they learn that I have HIV
  2. I worry that people who know I have HIV will tell others
  3. Most people with HIV are rejected when others find out
  4. Telling someone I have HIV is risky
  5. I will work hard to keep my HIV a secret
  6. People with HIV lose their jobs when their employers find out
  7. I don't feel ashamed of having HIV
  8. I will never feel the need to hide the fact that I have HIV
  9. Telling those close to you about my HIV means I will be able to receive more social support
  10. Telling those close to you about my HIV means I will have some to talk to about my feelings and problems
  11. If I tell someone about my HIV, I will be able to receive more help
  12. If I tell people about my HIV, I will be able to help others avoid getting HIV
