# Supplementary material for: Impact of Universal Test and Treat (UTT) on anticipated stigma among patients newly diagnosed with HIV in Johannesburg, South Africa: A cross-sectional study
Source: PLOS Glob Public Health. 2026 Jan 27;6(1):e0004779. doi: 10.1371/journal.pgph.0004779 (PMC12843591; doi:10.1371/journal.pgph.0004779)
Supplement: Attachment — Submitted filename: Response to reviewers.docx [file pgph.0004779.s004.docx]

1. Please provide additional details regarding participant consent. In the ethics statement in the Methods and online submission information, please ensure that you have specified (1) whether consent was informed and (2) what type you obtained (for instance, written or verbal, and if verbal, how it was documented and witnessed).

We thank you for this comment. We have provided details regarding consent and included these in the methods section.

2. We noticed you have some minor occurrence of overlapping text with the following previous publication(s), which needs to be addressed:

<https://www.tandfonline.com/doi/full/10.1080/09540121.2021.1902927>

We thank you for this comment and this suggestion. We have now explicitly referenced our previous publication, correctly cited it, and highlighted it more prominently, as the findings are consistent with the results reported in that study.

In your revision ensure you cite all your sources (including your own works), and quote or rephrase any duplicated text outside the methods section. Further consideration is dependent on these concerns being addressed.

3. In the online submission form, you indicated that [The de-identified data supporting the findings of this study are available from the corresponding author upon reasonable request, subject to ethical approvals and data use agreements in place.].

All PLOS journals now require all data underlying the findings described in their manuscript to be freely available to other researchers, either
a. In a public repository,
b. Within the manuscript itself, or
c. Uploaded as supplementary information.

This policy applies to all data except where public deposition would breach compliance with the protocol approved by your research ethics board. If your data cannot be made publicly available for ethical or legal reasons (e.g., public availability would compromise patient privacy), please explain your reasons by return email and your exemption request will be escalated to the editor for approval. Your exemption request will be handled independently and will not hold up the peer review process, but will need to be resolved should your manuscript be accepted for publication. One of the Editorial team will then be in touch if there are any issues.

We thank you for this comment. We have uploaded the data and code in a public repository with a link provided below.

(https://figshare.com/articles/dataset/Impact_of_Universal_Test_and_Treat_UTT_on_expected_stigma_among_patients_newly_diagnosed_with_HIV_in_Johannesburg_South_Africa_a_cross-sectional_study/30157579)

4. If the reviewer comments include a recommendation to cite specific previously published works, please review and evaluate these publications to determine whether they are relevant and should be cited. There is no requirement to cite these works unless the editor has indicated otherwise. 

Additional Editor Comments (if provided):

Dear authors,

Thank you for submitting your manuscript to PLOS Global Public Health. In addition to the reviewer comments, please address the following points:

1. Your cover letter describes a longitudinal cohort and impact/trends, but the manuscript and abstract describe a cross-sectional survey. Either (A) truly analyze pre- vs post-UTT with repeated measures/time trends or (B) reframe as prevalence and correlates in the UTT era. Suggested title: Anticipated HIV stigma among newly diagnosed adults in Johannesburg in the UTT era: a cross-sectional study.
We thank the reviewer for this detailed comment. We acknowledge that it was an oversight in the original manuscript. While the parent study was designed as a longitudinal cohort, the present analysis focuses specifically on cross-sectional data examining anticipated HIV stigma among newly diagnosed adults. We have revised the title accordingly. We have clarified the study design in the manuscript and revised the cover letter accordingly to accurately reflect the scope and nature of this analysis.

2. Methods: STROBE (cross-sectional) is the primary guideline for your study design; complete it, attach it as supplementary material and ensure complete reporting of design, setting, variables, bias, study size, statistical methods, and participant flow.

We thank the reviewer for this comment. We have completed and attached the STROBE form. We would also like to note that the CONSORT diagram from this study has already been published, and we therefore aim to avoid duplication.

3. Methods: Name and cite the five items used, show wording (in an appendix), and justify any adaptations.

We thank the reviewer for this comment. We have cited the sources for the five items used and included the justification for the adaptations. Our questionnaire included 12 questions to capture participants’ perceptions about the disclosure of HIV status. We then conducted a principal component analysis to examine whether these items measured a common underlying construct. The PCA identified five items that loaded strongly on a single factor and were retained to form the final variable. These items demonstrated good internal consistency, with a Cronbach’s alpha of 0.82, indicating that the 5 items reliably captured perceptions about disclosure. This is highlighted in the methods section, and we have added the questions in the appendix. (**Page 7, lines 151-160**)

4. Methods: Justify Modified Poisson with robust SE and state whether you clustered by clinic. It would also be important to pre-specify a confounder set (conceptual framework/DAG) and keeping it the same across models. How did you address missing data?

We thank the reviewer for this comment. We have justified the use of Modified Poisson with robust SE in the methods. The Modified Poisson regression approach used a robust variance estimator to directly estimate relative risks, and this is particularly useful for common conditions with a prevalence ≥10%, such as the anticipated stigma in this case, preventing odds ratios from accurately approximating RRs. We have added references to the methods section.

We decided not to cluster the Poisson regression by clinic because there are only five different clinics in the sample. When the number of clusters is this small, the cluster-robust standard errors become unstable and can give misleading results. Clustering usually works best when there are many clusters (generally 30 or more), as this allows for a more reliable adjustment. In our case, with 652 patients but only clinics, clustering would likely reduce the accuracy of the estimates rather than improve them. We have listed this as a limitation for our study.

Regarding the missing data, we compared the patient characteristics and found that the missing data were not systematic, but random. Additionally, <10 of % data was missing.

5. Results: You report lower anticipated stigma among those with low perceived social support—this likely reflects reverse coding or misclassification. Audit coding and table labels (e.g., “low vs high”).

We thank the reviewer for this comment. We have carefully re-examined the variables in question and confirm. We have confidence that the findings presented in the current analysis are robust and accurately reflect the underlying measurements. In the discussion, we explain why this may be the case.

**Page 24, lines 303-307:** *“Furthermore, those with low perceived social support were also less likely to report anticipated stigma. This could be as a result of them having developed coping mechanisms that help them handle stigma better and potential for alternative sources of validation. However, this finding should be interpreted with caution. Prior research shows that social support from friends and family can buffer against stigma and improve psychosocial outcomes, whereas family disapproval or conflict can exacerbate stigma and result in isolation. The interaction between social networks and anticipated stigma is complex and depends on the quality rather than the simple presence.”*

6. Results: Please double-check the “Primary house” categories and percentages (some proportions look implausible relative to the 55% overall).

We thank the reviewer for this comment. We have carefully re-examined the variables in question and confirm that the reported results have been validated in all prior publications from the main study. Table 1 presents column percentages for the overall sample (641/652 respondents), whereas Table 2 presents row percentages of participants with high anticipated stigma within each category. For example, 97 of 225 participants (43.5%) had high anticipated stigma.

Can the reviewer kindly clarify this question and whether this response is satisfactory?

7. Results: Ensure all variables mentioned (e.g., depression) are fully described in Methods (instrument, scoring, cut-points).

We thank the reviewer for this comment. We have provided further description of all the relevant variables and the corresponding measures used to measure the responses.

**Pages 7&8, lines 155-176** : “All questions were closed-ended with options For the final analysis, we dichotomized the final outcome of anticipated stigma whereby mean scores were categorized as "low-to-medium” (if the score<=2.5), or “high” (if the score>2.5)[27, 28] defining anticipated stigma with a four-point scale measuring agreement with statements regarding HIV disclosure concerns and HIV status concealment (Cronbach’s alpha =.82). Depression was measured using the CES-D 10 scale, a 10-question four-point scale (which had scores ranging from 0 to 3) that measures general depressive symptoms experienced up to seven days before the interview [29]. The total score ranged from zero to 30, with higher scores reflecting higher depression (Cronbach’s alpha = 0.81). Depression was dichotomized into major depression (total score of ≥ 12) and no major depression (total score < 12)[30, 31]. We created a household amenities index to measure participants’ access to 13 key amenities, including basic services (e.g., flush toilet, electricity, gas), housing quality, household density, food availability, and ownership of durable assets (e.g., TV, radio, refrigerator, phone, microwave, computer). The index showed good internal consistency (Cronbach’s alpha = 0.79)[32]. Concerns about ART were assessed using a 12-item, 4-point Likert scale questionnaire (Cronbach’s alpha = 0.83), covering worries about long-term effects, side effects, and perceived need for ART. Mean scores were categorized as low (<2), medium (2–3), or high (>3) concerns. Perceived social support (PSS) was measured with an 8-item satisfaction scale (Cronbach’s alpha = 0.61), and this measured participants’ satisfaction with the support made available to them [32], with mean scores categorized as low (<2), medium (2–<3), or high (≥3).”

Thank you,

Academic Editor Guillaume Fontaine


[Note: HTML markup is below. Please do not edit.]

Reviewers' comments:

Reviewer's Responses to Questions

**Comments to the Author**

1. Does this manuscript meet PLOS Global Public Health’s [publication criteria](https://track.editorialmanager.com/CL0/https:%2F%2Fjournals.plos.org%2Fglobalpublichealth%2Fs%2Fcriteria-for-publication/1/010f0198cc0404b0-7df4ca14-93bf-4881-b1bb-c90d5e44122b-000000/8PgVXh1QXiySsFTK6QzSFlhKf74B6sqf6pATk-Cybpc=225)? Is the manuscript technically sound, and do the data support the conclusions? The manuscript must describe methodologically and ethically rigorous research with conclusions that are appropriately drawn based on the data presented.

Reviewer #1: Yes

2. Has the statistical analysis been performed appropriately and rigorously?

Reviewer #1: Yes

3. Have the authors made all data underlying the findings in their manuscript fully available (please refer to the Data Availability Statement at the start of the manuscript PDF file)?

The [PLOS Data policy](https://track.editorialmanager.com/CL0/https:%2F%2Fjournals.plos.org%2Fglobalpublichealth%2Fs%2Fdata-availability/1/010f0198cc0404b0-7df4ca14-93bf-4881-b1bb-c90d5e44122b-000000/_JAIrZpGOCtYC8CbaHblZ0mwJLKXnotvuRSwCryMEWI=225) requires authors to make all data underlying the findings described in their manuscript fully available without restriction, with rare exception. The data should be provided as part of the manuscript or its supporting information, or deposited to a public repository. For example, in addition to summary statistics, the data points behind means, medians and variance measures should be available. If there are restrictions on publicly sharing data—e.g. participant privacy or use of data from a third party—those must be specified.

Reviewer #1: No

4. Is the manuscript presented in an intelligible fashion and written in standard English?

PLOS Global Public Health does not copyedit accepted manuscripts, so the language in submitted articles must be clear, correct, and unambiguous. Any typographical or grammatical errors should be corrected at revision, so please note any specific errors here.

Reviewer #1: Yes

5. Review Comments to the Author

Please use the space provided to explain your answers to the questions above. You may also include additional comments for the author, including concerns about dual publication, research ethics, or publication ethics. (Please upload your review as an attachment if it exceeds 20,000 characters)

Reviewer #1: Overall, a well-written study investigating levels of anticipated stigma in people newly diagnosed with HIV. The intro could be developed to elaborate on the novelty of the study. I have made some suggestions below around the interpretation of the findings and the development of the discussion

Abstract
Clarify in the methods that "low-to-medium” or “high” refers to anticipated stigma
It could be more intuitive to report high anticipated stigma was more associated with XYZ
Cronbach’s alpha reported in Abstract but not paper.

We thank the reviewer for this comment. The Cronbach alpha has been added to the main paper, and cut-off values have been defined in the methods section. (**Page 7, lines 158-160**)

Introduction
Lines 89-92 “The stigma index…” reference 8 does not correspond. Support the statement with a relevant and current reference. Expand on what internalised stigma means.

We thank the reviewer for this comment. We have revised the sentence and also expanded on the meaning of internalised stigma

**Page 4, lines 91-94:***“Internalised stigma, characterised by feelings of low self-worth, guilt, and shame, persists* *[8] . It often results in fear of disclosure, self-blame, and recent reports have revealed an overall pooled prevalence to be 35.7% among people living with HIV in an African setting [10]”*

Line 103 – clarify if it refers to poor adherence to ART

We thank the reviewer for this comment. We have added ART at the end of the sentence:

**Page 5, line 103** *“Among other factors, anticipated stigma is a significant predictor of poor adherence to ART [16-18].”*

Line 115 – explain the changes to treatment guidelines

We thank the reviewer for this comment. The paragraph has been expanded to explain the changes in treatment guidelines.

**Page 5, lines 115-120:** *“These include evidence-based interventions, community engagement, and collaborations between different sectors. In light of the changes in treatment guidelines which include the country progressively raising the CD4-based ART eligibility threshold from 500 cells/µL (2015) and September 2016, it removed the CD4 threshold entirely and adopted the WHO Universal Test and Treat (UTT) policy, making all people living with HIV eligible for ART immediately upon diagnosis[3, 4], we aimed to determine the prevalence and predictors of anticipated stigma among newly HIV-diagnosed individuals under the UTT policy in Johannesburg, South Africa.”*

Line 116 – UTT has not been previously spelt out/mentioned/ explained

We thank the reviewer for this comment. Universal Test and Treat (UTT) has been spelled out prior to use of the acronym.

**Page 5, line 119:***“…., it removed the CD4 threshold entirely and adopted the WHO Universal Test and Treat (UTT) policy, making all people living with HIV eligible for ART immediately upon diagnosis[3, 4]”*

Introduction needs a further paragraph explaining the need for this study. I think the novelty lies in providing updated data in response to the National Strategic Plan and could provide more information on the South African context of UTT etc. Could cut some of the introduction which is repetitive e.g., Lines 108-111

We thank the reviewer for this suggestion. We have added a new paragraph providing further justification.

**Pages 5-6, lines 126-129:** “*Understanding the prevalence and predictors of anticipated stigma in this context is therefore crucial for informing targeted interventions, supporting treatment adherence, and achieving national HIV targets. A study in Umlazi Township found that 30.8% of participants reported anticipated HIV stigma, with higher prevalence among women and individuals living with HIV[25]. This study aims to provide data on anticipated stigma in Johannesburg, thus contributing to the evidence base needed to inform stigma-reduction strategies and improve HIV care outcomes in line with the National Strategic Plan's objectives”*

Methods
Expand on recruitment methods: location, how many sites, what type of sites

We thank the reviewer for this suggestion. We have expanded in the methods section.

**Page 6, line 132-133*:****“The study enrolled newly diagnosed adult participants (≥18 years) from October 2017 to August 2018 at four primary healthcare clinics (PHCs) in Johannesburg, South Africa.”*

Lines 128-130 delete description as it is stated in Results

We thank the reviewer for this suggestion. We have deleted the repetitive description

Line 132 it sounds like a five point scale was used

We thank the reviewer for this comment. This has been rectified, it is 4-point Likert scale. (**Page 7, line 151)**

Results
Not clear why Table 1 is stratified by sex when there is no further stratified analysis. Add justification (in the introduction and/or methods) or just present the overall sample characteristics

We thank the reviewer for this comment. We have presented the overall sample characteristics. In our previous publications, Table 1 was presented in the same manner; however, in the current analysis, there is no justification for doing so.

Line 159 – says characteristics are presented by gender but the text presents by sex male/female

We thank the reviewer for this comment. We have rectified this to reflect. (**Page 9, line 198**)

Line 161 – add time period of recruitment and information on number of recruitment sites

We thank the reviewer for this comment. We have added the time period of recruitment and the number of sites in the first line of the methods section.

**Page 6, line 132-133:**““*The study enrolled newly diagnosed adult participants (≥18 years) from October 2017 to August 2018 at four primary healthcare clinics (PHCs) in Johannesburg, South Africa.”*

Table 2 presents the same data as Figure 1. Might be better to replace Figure 1 with a figure summarizing the mean score response to each of the five questions. Also provide the five questions in full
We thank the reviewer for this comment. We have created box plots that illustrate the median scores of anticipated stigma. However, they did not show a clear distinction between high and low-to-medium prevalence. In Figure 1, the outcome of anticipated stigma was dichotomized, with mean scores classified as “low-to-medium” (≤2.5) or “high” (>2.5). This is the same categorisation applied in the final model using the same variable in Figure 1. Would it be okay with the reviewer if we kept Figure 1 instead?

Discussion
Line 220 – not clear on the connection between anticipated stigma and receiving counselling

We thank the reviewer for this comment. Our understanding is that high quality post-test counselling can reduce fears of stigma and discrimination as it addresses plans around disclosure, myths about HIV, and ART coping strategies. However, in some instances, stigma persists. We have removed the latter part of the sentence.

Line 227 – add reference

We thank the reviewer for this comment. We have added a reference that speaks about the moral judgments and the differences by sex.

Lines 234-248 in this section I would just bear in mind that the aRR is just on the cusp of significance, so in the last sentence I don’t think your findings contrast with previous because there is no statistically significant difference between married and single people in this study

We thank the reviewer for this comment. We have carefully considered the feedback and revised the text accordingly. Specifically, we have refrained from drawing strong conclusions and have removed the final sentence that stated our results were in contrast to previous reports, as our findings are on the cusp of statistical significance.

Lines 249-256 could explore whether younger people having more anticipated stigma is related to them having more potential partners. Then discuss in relation to raising awareness of U=U

We thank the reviewer for this suggestion. We have expanded the discussion to explore whether higher levels of anticipated stigma among younger individuals may be partly explained by their greater likelihood of having more potential sexual partners and concerns about disclosure in new relationships.

**Page 24, lines 303-307:***“Furthermore, young people are more likely to get into more relationships and have more potential sexual partners, and may experience more fears of disclosure and may amplify anticipated stigma due to worries about rejection, and relationships coming to an end upon disclosure[48]. This underscores the importance of targeted messaging around the Undetectable = Untransmittable (U=U), which has shown potential to reduce stigma [49].”*

Line 257-261 Could this finding be related to the questions – people aren’t concerned about being rejected/judged because they don’t have friends/family around them? I think there needs to be a more nuanced interpretation here – considering that support from friends/family might counter some types of stigma but disapproval (?) from family can be isolating

We thank the reviewer for this comment, we have expanded and provided a more nuanced interpretation.,

**Pages 24-25, lines 313-318**: *“Furthermore, those with low perceived social support were also less likely to report anticipated stigma. This could be as a result of them having developed coping mechanisms that help them handle stigma better and potential for alternative sources of validation. However, this finding should be interpreted with caution. Prior research shows that social support from friends and family can buffer against stigma and improve psychosocial outcomes, whereas family disapproval or conflict can exacerbate stigma and result in isolation. [51, 52]. The interaction between social networks and anticipated stigma is complex and depends on the quality rather than simple presence.”*

Could add a paragraph on policy implications – what can be done to address high prevalence of anticipated stigma

We thank the reviewer for this comment. We have added a paragraph that speaks to the policy implications

**Page 25, lines 322-326*:****““High levels of stigma necessitate comprehensive interventions that address psychosocial and structural determinants of stigma. Evidence underscores the effectiveness of community-based and culturally appropriate interventions, the integration of U=U, and the strengthening of anti-discrimination laws, and actively engaging PLHIV when designing interventions to mitigate stigma.”*

Minor
Spell out universal test and treat in the title

We thank the reviewer for this comment. We have spelled out UTT in the title

Some typos – capitalize first letter of background paragraph in abstract

We thank the reviewer for this comment. The “A” in anticipated stigma in the background paragraph in the abstract is capitalised.

Spell out acronyms which are lesser used like “National Strategic Plan”

We thank the reviewer for this comment, and we have ensured that all acronyms are spelled out including the National Strategic Plan.
